# Rapalink-1 Increased Infarct Size in Early Cerebral Ischemia–Reperfusion With Increased Blood–Brain Barrier Disruption

**DOI:** 10.3389/fphys.2021.706528

**Published:** 2021-07-20

**Authors:** Oak Z. Chi, Xia Liu, Sean Cofano, Nikhil Patel, Estela Jacinto, Harvey R. Weiss

**Affiliations:** ^1^Department of Anesthesiology and Perioperative Medicine, Rutgers Robert Wood Johnson Medical School, New Brunswick, NJ, United States; ^2^Department of Neuroscience and Cell Biology, Rutgers Robert Wood Johnson Medical School, Piscataway, NJ, United States; ^3^Department of Biochemistry and Molecular Biology, Rutgers Robert Wood Johnson Medical School, Piscataway, NJ, United States

**Keywords:** blood-brain barrer, brain protection, cerebral ischemia-reperfusion, mTOR inhibitor, Rapalink-1, ^14^C-α-aminoisobutyric acid

## Abstract

It has been reported that the mechanistic target of rapamycin (mTOR) pathway is involved in cerebral ischemia–reperfusion injury. One of the important pathological changes during reperfusion after cerebral ischemia is disruption of blood–brain barrier (BBB). Rapamycin, a first-generation mTOR inhibitor, produces divergent effects on neuronal survival and alteration in BBB disruption. In this study, we investigated how Rapalink-1, a third-generation mTOR inhibitor, would affect neuronal survival and BBB disruption in the very early stage of cerebral ischemia–reperfusion that is within the time window of thrombolysis therapy. The middle cerebral artery occlusion (MCAO) was performed in rats under isoflurane anesthesia with controlled ventilation. Of note, 2 mg/kg of Rapalink-1 or vehicle was administered intraperitoneally 10 min after MCAO. After 1 h of MCAO and 2 h of reperfusion, the transfer coefficient (K_i_) of ^14^C-α-aminoisobutyric acid (104 Da) and the volume of ^3^H-dextran (70,000 Da) distribution were determined to assess the degree of BBB disruption. At the same time points, phosphorylated S6 (Ser240/244) and Akt (Ser473) as well as matrix metalloproteinase-2 (MMP2) protein level were determined by Western blot along with the infarct size using tetrazolium stain. Rapalink-1 increased the K_i_ in the ischemic-reperfused cortex (IR-C, +23%, *p* < 0.05) without a significant change in the volume of dextran distribution. Rapalink-1 increased the percentage of cortical infarct out of the total cortical area (+41%, *p* < 0.005). Rapalink-1 significantly decreased phosphorylated S6 and Akt to half the level of the control rats in the IR-C, which suggests that both of the mechanistic target of rapamycin complex 1 and 2 (mTORC1 and mTORC2) were inhibited. The MMP2 level was increased suggesting that BBB disruption could be aggravated by Rapalink-1. Taken together, our data suggest that inhibiting both mTORC1 and mTORC2 by Rapalink-1 could worsen the neuronal damage in the early stage of cerebral ischemia–reperfusion and that the aggravation of BBB disruption could be one of the contributing factors.

## Introduction

Within the first few hours after the onset of stroke, despite the return of blood flow with or without tissue plasminogen activator (tPA) or endovascular thrombolysis, ischemia–reperfusion injury and neuronal damage may result in multiple pathological events such as excitotoxicity, necrosis, oxidative stress, inflammation, apoptosis, platelet adhesion, and blood–brain barrier (BBB) disruption (Lin et al., 2016; Mayor and Tymianski, 2018; Andjelkovic et al., 2019).

Mechanistic target of rapamycin (mTOR), as a catalytic subunit of two distinct protein complexes, namely, rapamycin-sensitive mTORC1 and rapamycin-insensitive mTORC2, is a protein kinase and an integral regulator of cellular proliferation, apoptosis, growth, metabolism, and autophagy (Murugan, 2019; Szwed et al., 2021). Both mTORCs are involved in maintaining metabolic homeostasis by responding to intracellular and environmental nutrient conditions. Recently, we have shown that the inhibition or activation of the various proteins or kinases involved in mTOR pathways has been associated with neuronal survival and BBB disruption during the early stage of ischemia–reperfusion, especially within the time window of thrombolysis therapy (Chi et al., 2016a,b; Liu et al., 2018; Chi et al., 2019), which is 3–4.5 h for tPA treatment and 6 h for endovascular thrombectomy after stroke (Boulanger et al., 2018). Rapalink-1 is a third-generation mTOR inhibitor comprising rapamycin and a second-generation ATP-competitive mTOR kinase inhibitor (TORKI). It ablates the activity of both multiproteins mTORC1 and mTORC2, but it has a more durable effect than conventional TORKI (Rodrik-Outmezguine et al., 2016; La Manna et al., 2020).

In spite of the involvement of mTORC1 and mTORC2 in cell survival pathways, the effects of individual mTORC1 or mTORC2 on neuronal survival in cerebral ischemia–reperfusion are limited since their effectors and pathways are interconnected to each other. Using rapamycin, a first-generation mTOR inhibitor, we have demonstrated the aggravation of cerebral ischemia at 2 h of reperfusion after 1 h of middle cerebral artery occlusion (MCAO) (Chi et al., 2016a). In that experiment, the phosphorylation of S6 (i.e., Ser240/244) and the phosphorylation of Akt (i.e., Ser473) were determined for mTORC1 and mTORC2 activity, respectively. The activation of both mTORC1 and mTORC2 was enhanced by ischemia–reperfusion, and rapamycin significantly inhibited both mTORC1 and mTORC2 in the ischemic-reperfused cortex (IR-C). Our data suggest that blocking both mTORC1 and mTORC2 is detrimental to neurons in the first few hours of cerebral ischemia–reperfusion. In contrast, in other studies with rapamycin, possible neuroprotection in cerebral ischemia–reperfusion was reported (Fletcher et al., 2013; Guo et al., 2014; Yang et al., 2019; Beard et al., 2020). In most of these studies, the activity of mTORC1 or mTORC2 in cerebral ischemia–reperfusion was not reported and their data were collected in the later stage of cerebral ischemia–reperfusion.

One of the important pathophysiological changes in cerebral ischemia–reperfusion is BBB disruption. BBB is a part of the neurovascular unit at the capillary level, which is anatomical and functional (McConnell et al., 2017; Yu et al., 2020). It is composed of endothelium, pericytes, astrocytes, neurons, tight junctions, and basal lamina. Poststroke BBB disruption appears to be predictive of functional outcome irrespective of stroke size (Andjelkovic et al., 2019; Nadareishvili et al., 2019). After thrombolytic treatment for stroke, the risk of hemorrhagic complication is increased with BBB disruption (Butler et al., 2020). It has been emphasized that BBB protection is one of the therapeutic strategies for acute ischemic stroke (Sifat et al., 2017).

There are studies suggesting the involvement of mTOR pathway in altering BBB disruption in cerebral ischemia–reperfusion. The activation of mTOR pathways has been associated with enhancing hypoxia-inducible factor 1-alpha (HIF-1α), vascular endothelial growth factor (VEGF), and matrix metalloproteinases (MMPs), which is related to increase BBB permeability in acute cerebral ischemia (Land and Tee, 2007; Shen et al., 2018; Murugan, 2019). The inhibition of p70 ribosomal S6 kinase-1 (S6K1) by PF-4708671 decreased infarct size with decreased BBB disruption in early cerebral ischemia–reperfusion (Chi et al., 2019). Guo et al. reported that rapamycin decreased infarct volume with decreased brain edema (Guo et al., 2014). Activated Akt increased BBB disruption but reduced infarct size (Weiss et al., 2018). But rapamycin decreased neuronal survival despite decreased BBB disruption (Chi et al., 2016a,b). Collectively, the outcome of neuronal survival with the modification of mTOR pathway in cerebral ischemia–reperfusion could depend on its net effects on neurons and the BBB.

In this study, we investigated whether Rapalink-1, a third-generation mTOR inhibitor, would inhibit both mTORC1 and mTORC2 and its effects on neuronal survival and BBB disruption in cerebral ischemia–reperfusion. At 2 h after 1 h of MCAO, the transfer coefficient (K_i_) of ^14^C-α-aminoisobutyric acid (^14^C-AIB) and the volume of ^3^H-dextran (70,000 Da) were determined to assess the functional degree of BBB disruption. The size of the infarct was determined with tetrazolium staining. The phosphorylation of S6 (i.e., Ser240/244) and the phosphorylation of Akt (i.e., Ser473) were determined to assess the activity of mTORC1 and mTORC2, respectively. To investigate the molecular mechanism of BBB disruption, the level of the matrix metalloproteinase-2 (MMP2) protein, which is a collagenase and degrades tight junction and basement membrane of the BBB, was determined (Liu et al., 2012; Huber et al., 2019).

We found that Rapalink-1 inhibited both mTORC1 and mTORC2 and increased infarct size along with increased BBB disruption in the first few hours of cerebral ischemia–reperfusion. Our data suggest that Rapalink-1-mediated dual inhibition of mTORC1 and mTORC2 could be detrimental to neuronal survival during the early stage of cerebral ischemia–reperfusion, especially within the time window of thrombolysis therapy. The increased BBB disruption could be one of the contributing factors for increased infarct size.

## Materials and Methods

### Animals

We followed the US Public Health Service Guidelines and the Guide for the Care of Laboratory Animals (DHHS Publication No. 85-23, revised 1996) in this study. We also obtained approval from our Institutional Animal Care and Use Committee.

A total of 38 male Fischer 344 rats weighing 220–250 g were used. They were randomly divided into two groups, i.e., 19 rats in each group: (1) control group (MCAO/reperfusion) and (2) Rapalink-1 group (MCAO/reperfusion + Rapalink-1). For the Rapalink-1 group, 2 mg/kg of Rapalink-1 dissolved in a solution of 5% DMSO, 5% TWEEN 80, and 5% PEG 350 in distilled water (Rapalink-1 concentration: 2 mg/10 ml) was administered intraperitoneally 10 min after transient MCAO. The dose of Rapalink-1 used in this study was similar to the dose used to decrease alcohol intake in mice (Morisot et al., 2018). For the control group, at the same time points, the same volume of vehicle was administered. All rats were ventilated through the tracheal tubes with 2% isoflurane in an air–oxygen mixture for MCAO. The isoflurane concentration was maintained at 1.4% after MCAO. A femoral arterial catheter was inserted to connect to the Statham P23Db pressure transducer, an Iworx data acquisition system (Dover, NH, USA) was used to monitor heart rate and blood pressure, and a Radiometer blood gas analyzer (ABL80, Brea, CA, USA) was used to obtain blood samples for the analysis of hemoglobin, blood gases, and pH. A femoral venous catheter was used to administer radioactive tracer and normal saline. Body temperature was monitored with a servo-controlled rectal thermistor probe. It was maintained at 37 ± 0.5°C with a heating lamp. As a representative pericranial temperature, temporalis muscle temperature was monitored using a thermocouple probe (Omega Engineering, Inc., Stamford, CT, USA), which was 36.8 ± 0.4°C.

### Transient Middle Cerebral Artery Occlusion

To study cerebral ischemia–reperfusion, we performed transient MCAO by using an intraluminal thread (Longa et al., 1989; Chi et al., 2016a). Through a midline ventral cervical incision, the common carotid artery was exposed and was carefully separated from the adjacent nerve. A 4.0 monofilament thread with a silicone-covered tip was inserted into the stump of the external carotid artery and advanced ~1.7 cm into the internal carotid artery until the resistance was met. The filament was kept in place for 60 min to induce MCAO. Then, it was removed to allow reperfusion, and the external carotid artery was closed. All measurements were performed after 2 h of reperfusion. The BBB permeability parameters were determined in the ischemic-reperfused cortex (IR-C), contralateral cortex (CC), ipsilateral hippocampus (IH), contralateral hippocampus (CH), cerebellum (CBLL), and pons. Total surgical time including vascular cannulations and transient MCAO was almost identical for all the experimental animals.

### Blood–Brain Barrier Permeability

In 10 rats in each group, after 1 h of MCAO and 2 h of reperfusion, to determine BBB permeability, 20 μCi of ^14^C-AIB (molecular weight of 104 Da, Amersham, Arlington Heights, IL, USA) was rapidly injected intravenously and flushed with 0.5 ml of normal saline as previously described (Gross et al., 1987; Chi et al., 2016b). Blood samples were collected from the femoral arterial catheter at 20-s intervals for the first 2 min and then at every minute for the next 8 min. Five min after injecting ^14^C-AIB, 20 μCi of ^3^H-dextran (molecular weight of 70,000 Da, Amersham, Arlington Heights, IL, USA) was injected intravenously and flushed with 0.5 ml of normal saline. After collecting the 10-min arterial blood sample, the animals were decapitated. The following brain regions were dissected: IR-C, CC, IH, CH, CBLL, and pons. For the IR-C, we dissected the temporoparietal cortex about 4 mm in diameter and 2 mm in thickness, directly within the perfusion field of the MCA. We did not differentiate between ischemic core and penumbra area. For the CC, we dissected the opposite corresponding cortical area. The brain samples were solubilized in Soluene™ (Packard, Downers Grove, IL, USA). The arterial blood samples were centrifuged, and the plasma was separated. The plasma and brain samples were counted on a liquid scintillation counter that was equipped for dual label counting. Quench curves were prepared using carbon tetrachloride. All samples were automatically corrected for quenching. The blood–brain K_i_ for ^14^C-AIB was determined by assuming a unidirectional transfer of ^14^C-AIB over a 10-min period of the experiment using the following equation as used previously (Gross et al., 1987; Chi et al., 2016b):

$$\begin{array}{l} K_{i}=\frac{Am-\left(Vp\times CT\right)}{\int_{\;o}^{\;T}Cp\left(t\right)dt} \end{array}$$

where Am is the amount of ^14^C-AIB radioactivity in the tissue per gram and Vp is the volume of plasma retained in the tissue. Vp is determined from the ^3^H-dextran data and the following equation: Vp = A′m/C′p, where A′m is the amount of ^3^H-dextran radioactivity in the tissue per gram and C′p is the concentration of ^3^H-dextran in the plasma at the time of decapitation. Cp(*t*) is the arterial concentration of ^14^C-AIB over time *t*, and CT is the arterial plasma concentration of ^14^C-AIB at the time of decapitation. In the equation used to determine K_i_, Vp × CT is a correction term that accounts for the label ^14^C retained in the vascular compartment of the tissue, Am.

### Size of Infarction

In six rats in each group, to determine the size of the cortical infarct, the tetrazolium staining was performed. Of note, 0.05% of 2,3,5-triphenyltetrazolium chloride (Sigma-Aldrich, MO, USA) solution in phosphate-buffered saline (PBS) was prepared and warmed to 37°C (Joshi et al., 2004). After removing the brain from the head, it was sliced in coronal sections using a straight-edged razor blade. Typically, from each brain, this method yielded 3–4 slices that were ~2–3-mm thick each. The slices were placed in the tetrazolium for 30-min incubation. Then, tetrazolium was poured off, and the slices were washed three times in PBS, i.e., 1 min per wash. Each slice was then placed in a small weighing boat. To keep the slice from drying, the boat was prefilled with PBS. The boat was placed on a dissection microscope, and a clean slide was placed over it. The cortical region of each slice was traced onto the slide using a 0.3-mm marker. Any infarcted areas were marked by crosshatching over any areas that were not well-marked with tetrazolium stain. The slides were scanned, and the scanned images were measured for the total and infarcted cortical area by using the ImageJ software (National Institutes of Health, MD, USA), enabling to show the percentage of infarcted cortical area out of the total cortical area.

### Western Blot

In three rats of the control group (MCAO/reperfusion) and of the Rapalink-1 group (MCAO/reperfusion + Rapalink-1), brain tissue from the IR-C and CC was lysed in a radioimmunoprecipitation assay buffer (RIPA buffer), which is made of 150 mM NaCl, 25 mM Tris–HCl, pH 7.4, 1% NP-40, 0.25% sodium deoxycholate, 1 mM EDTA, 1 mM Na_3_PO_4_, and 1 mM NaF, with protease and phosphatase inhibitor cocktail. The tissue lysate was centrifuged at 15,000 × *g* for 30 min at 4°C. To quantify the protein levels and to normalize the sample concentrations to 1–5 mg/ml, the Bradford assay was used. In each lane, 20–30 μg of total extracts were loaded, and proteins were resolved by SDS–PAGE followed by the incubation of primary antibody (i.e., 1:1,000 overnight at 4°C) and secondary antibody (i.e., 1:5,000 1 h at room temperature) to detect phosphoproteins and total proteins. Antibodies such as S6, pS6 (i.e., Ser240/244), Akt, and pAkt (i.e., Ser473) were purchased from Cell Signaling (Danvers, MA, USA), and MMP2 from Abcam (Cambridge, MA, USA).

### Statistical Analysis

A two-way ANOVA was performed using the general linear model (i.e., PROG GLM) from the SAS Institute (Cary, NC, USA) to assess the differences in K_i_, the volume of dextran distribution, and the vital signs between the experimental groups and among the various examined regions. The statistical significance of differences was determined using the Tukey's test. The differences in the size of cortical infarct were analyzed with an unpaired Student's *t*-test. For the quantification of protein, ANOVA followed by multiple comparisons with Bonferroni correction was used. All data were expressed as mean ± SD, and the significance was defined as *p* < 0.05.

## Results

### Hemodynamic Parameters and Blood Gases

Hemodynamic variables and blood gases of the control and the Rapalink-1-treated groups prior to measuring BBB permeability at 2 h of reperfusion after 1 h of MCAO are presented in Table 1. Their values are within normal ranges for anesthetized rats. There were no statistically significant differences in any of the parameters between the Rapalink-1-treated and Rapalink-1-untreated rats. In the rats used for the determination of the size of cortical infarct and in the rats used for Western blot, the vital signs were similar and blood gases of the rats were maintained at similar values with controlled ventilation to the corresponding group of the rats used to determine BBB permeability.

**Table 1:** Hemodynamic and blood gas parameters for the control and the Rapalink-1-treated group at 2 h of reperfusion and just before the determination of blood–brain barrier permeability.

**Group**	**Control group (MCAO/reperfusion)**	**Rapalink-1 group (MCAO/reperfusion + Rapalink-1)**
Mean blood pressure (mmHg)	95 ± 13	97 ± 19
Heart rate (beats/min)	335 ± 28	350 ± 44
Arterial PO_2_ (mmHg)	110 ± 19	108 ± 19
Arterial PCO_2_ (mmHg)	33 ± 9	35 ± 9
pH	7.36 ± 0.06	7.34 ± 0.09
Hemoglobin (g/100 ml)	10.8 ± 0.9	11.1 ± 1.9

*MCAO, middle cerebral artery occlusion. Values are mean ± SD*.

### Transfer Coefficient (K_i_)

**Figure 1:**
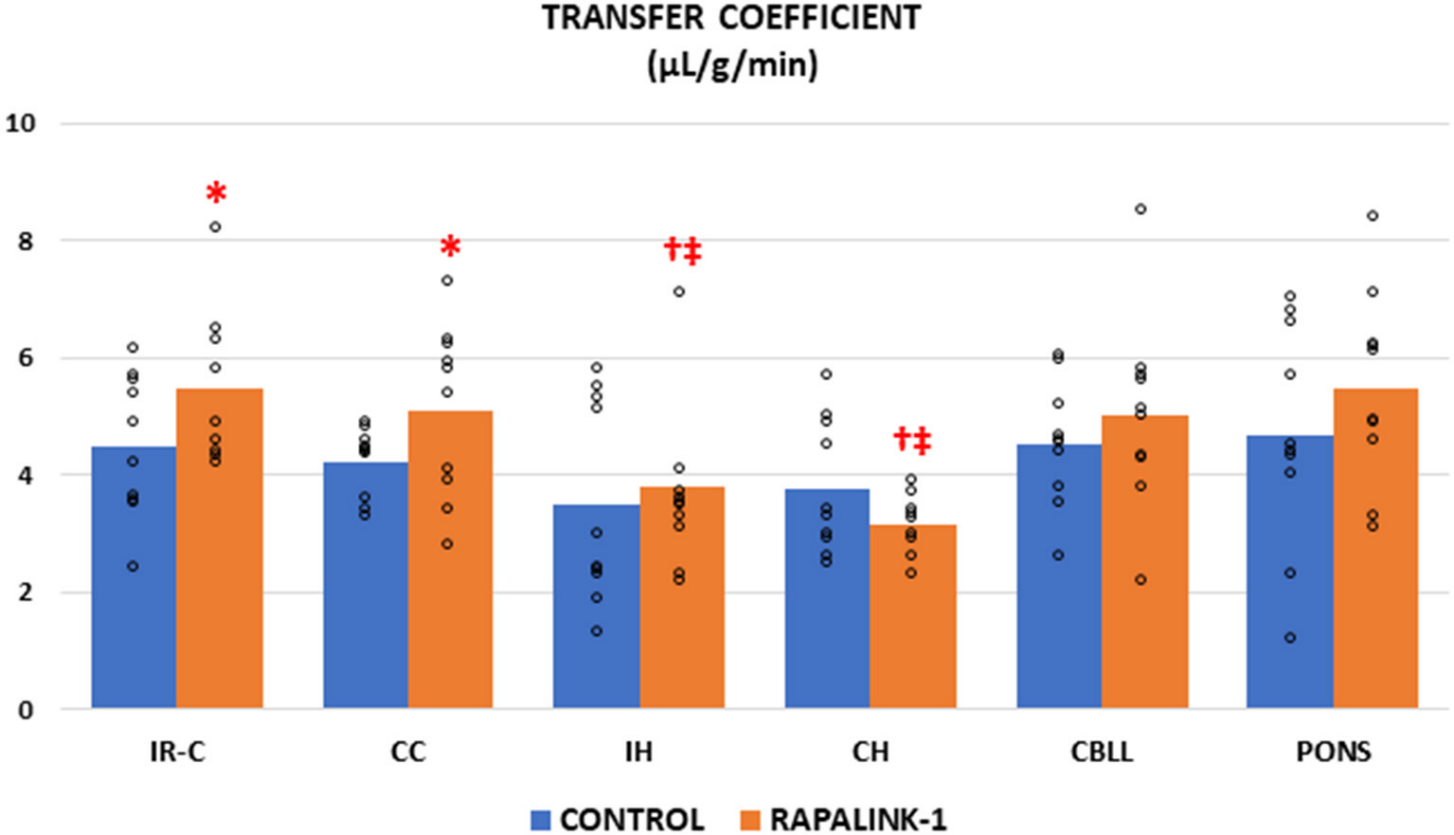
Dot plots for the transfer coefficient (K_i_) of ^14^C-AIB in the examined brain regions of the control group [middle cerebral artery occlusion (MCAO)/reperfusion] and the Rapalink-1 group (MCAO/reperfusion + Rapalink-1) after 1 h of MCAO and 2 h of reperfusion. Each bar represents the mean value of each brain region. A two-way ANOVA followed by the Tukey's test for multiple comparisons was used. The K_i_ for ^14^C-AIB was increased with Rapalink-1 in the IR-C and in the CC. *n* = 10 in each group. IR-C, ischemic-reperfused cortex; CC, contralateral cortex; IH, ipsilateral hippocampus; CH, contralateral hippocampus; CBLL, cerebellum. *p < 0.05 vs. the control group (MCAO/reperfusion). ^†^*p* < 0.05 vs. pons. ^‡^*p* < 0.05 vs. IR-C.

The administration of Rapalink-1 increased the K_i_ of ^14^C-AIB in the IR-C [+23%, *F*_(1, 17)_ = 4.58, *p* < 0.05]. In most of the non-ischemic brain regions, the K_i_ appeared higher with Rapalink-1 treatment but with a statistical significance only in the CC [*F*_(1, 18)_ = 4.50, *p* < 0.05]. The K_i_ of all other non-ischemic brain regions was similar to the CC in the control group. In the Rapalink-1 group, however, the K_i_ of the IH and CH were lower than the IR-C and the pons [*F*_(5, 52)_ = 4.01, *p* < 0.01] (Figure 1).

### Volume of Dextran Distribution

**Figure 2:**
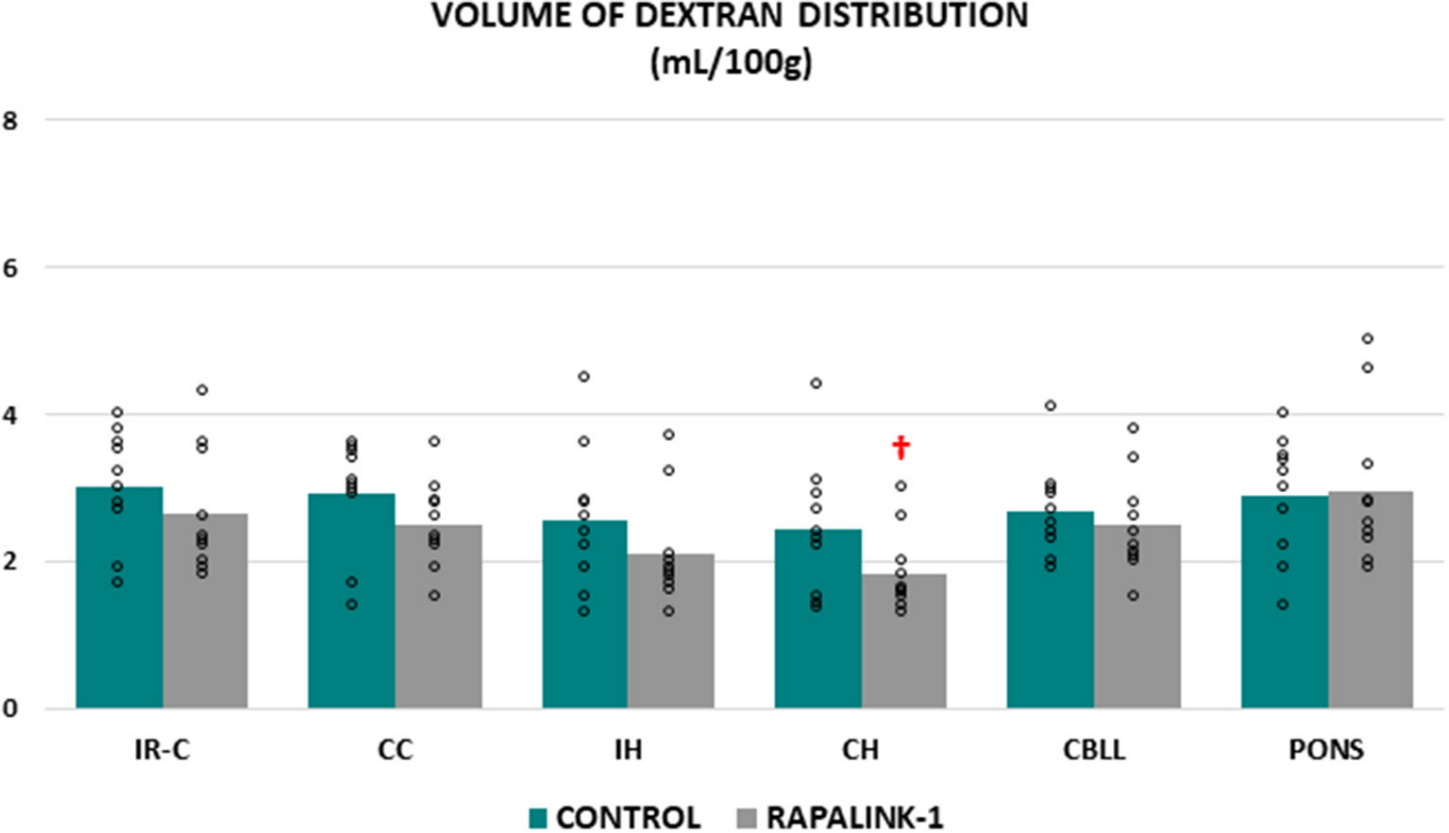
Dot plots for the volume of dextran distribution in the examined brain regions of the control group (MCAO/reperfusion) and the Rapalink-1 group (MCAO/reperfusion + Rapalink-1) after 1 h of MCAO and 2 h of reperfusion. Each bar represents the mean value of each brain region. A two-way ANOVA followed by the Tukey's test for multiple comparisons was used. There were no significant changes in the volume of dextran distribution with Rapalink-1 in every brain region that was studied. IR-C, ischemic-reperfused cortex; CC, contralateral cortex; IH, ipsilateral hippocampus; CH, contralateral hippocampus; CBLL, cerebellum. *n* = 10 in each group. ^†^*p* < 0.05 vs. pons.

There was no significant difference in the volume of dextran distribution between the control group and the Rapalink-1 group in any brain region, even though it appeared lower in the Rapalink-1 group in most of the brain regions. In each group, there was no significant difference in the volume of dextran distribution among the brain regions except that it was lower in the CH than the pons [*F*_(5, 54)_ = 2.68, *p* < 0.05] in the Rapalink-1 group (Figure 2).

### Size of Infarction

**Figure 3:**
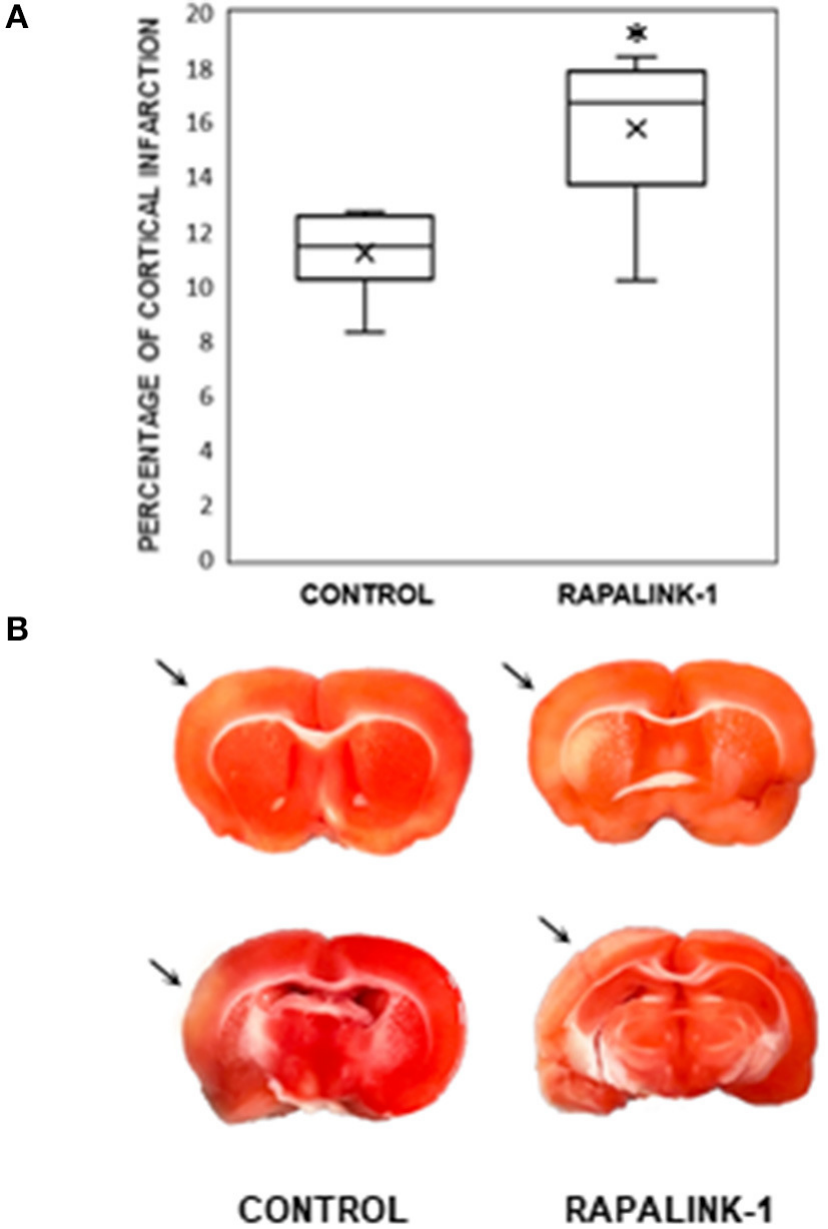
**(A)** A diagram with box plots showing the percentage of cortical infarcted area compared with total cortical area in the experimental groups after 1 h of MCAO and 2 h of reperfusion. Each boxplot consists of 25th percentile, median, 75th percentile, and whiskers with the minimum and maximum. Mean is shown with the symbol “×.” Rapalink-1 increased the percentage of the cortical infarct out of the total cortical area. *n* = 6 in each group. An unpaired Student's *t*-test was used for the significance. *p < 0.005 vs. the control group (MCAO/reperfusion). **(B)** Two representative brain sections from the similar brain regions of the control group (MCAO/reperfusion) and the Rapalink-1 group (MCAO/reperfusion + Rapalink-1). The tissue samples were photographed at 2 h after reperfusion without any fixatives. The area of the infarcted cortex appeared pink, not white, which suggests infarction is still going on. The infarcted cortices are marked with arrows. Some of the subcortical areas appeared to be infarcted.

An infarcted area was observed in the affected cortex of each rat in both groups. The percentage of cortical infarct area out of the total cortical area was significantly increased in the Rapalink-1 group than the control group (+41%, *p* < 0.005, Figure 3).

### Western Blot

**Figure 4:**
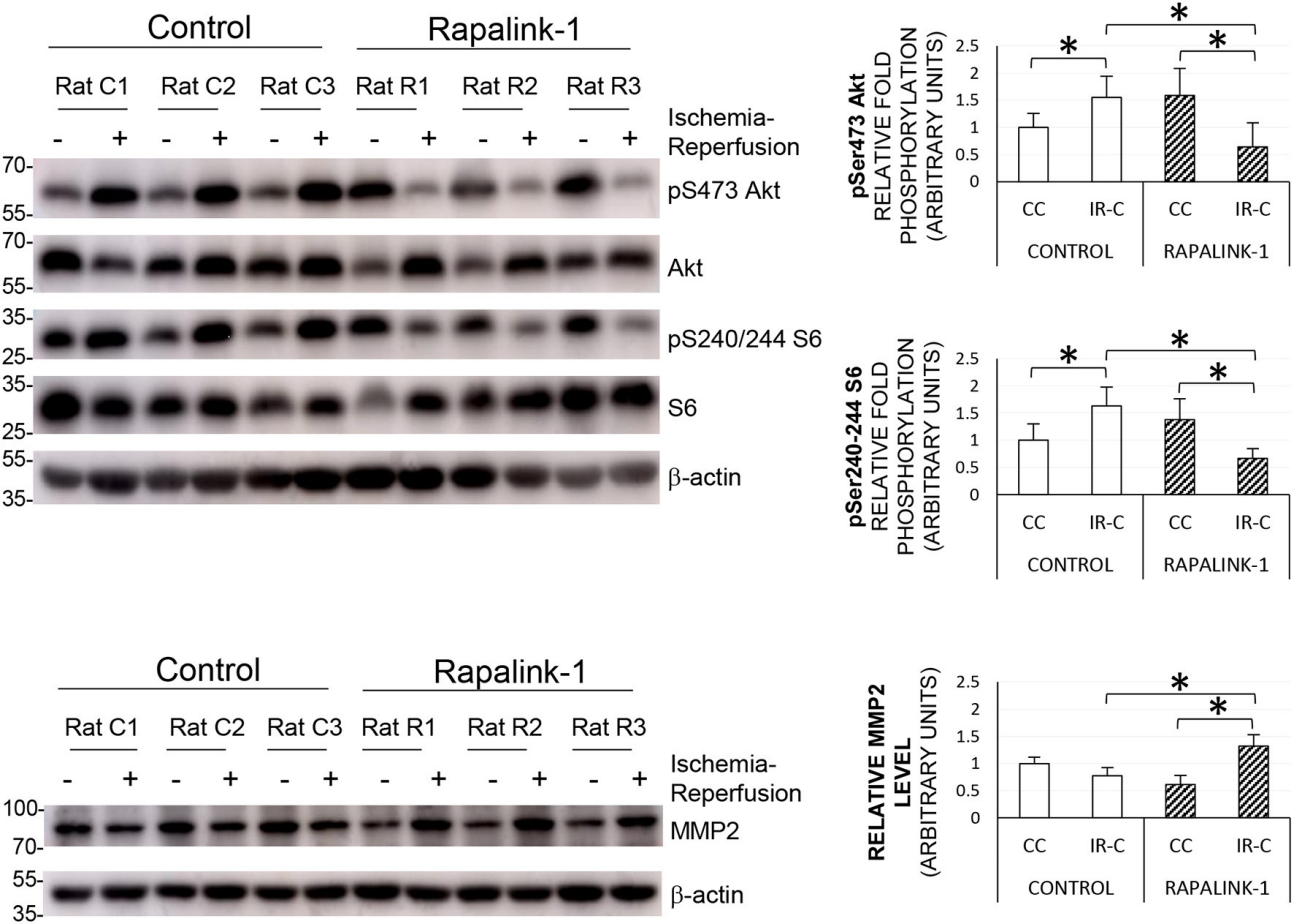
The representative Western blots of pS6, pAkt, and MMP2, and their quantification after 1 h of MCAO and 2 h of reperfusion. ANOVA followed by multiple comparisons with the Bonferroni correction was used for the quantification of protein. The Phosphorylation of Akt at Ser473 and the phosphorylation of S6 at Ser240/244 were increased during ischemia–reperfusion that indicates the increased activity of mTORC2 and mTORC1, respectively. The treatment with Rapalink-1 resulted in a more than 50% decrease in the phosphorylation of both Akt and S6 in the IR-C when compared with the control rats, which suggests that Rapalink-1 inhibited both mTORC1 and mTORC2 in this experiment. The increased protein level of MMP2 with Rapalink-1 treatment in the IR-C suggests that Rapalink-1 could aggravate blood–brain barrier (BBB) disruption in the ischemic-reperfused area. CC, contralateral cortex; IR-C, ischemic-reperfused cortex; Control, MCAO/reperfusion group; Rapalink-1, MCAO/reperfusion + Rapalink-1 group. *n* = 6–9. *p < 0.05. Values are means ± SD.

To validate the inhibition of both mTORC1 and mTORC2 by Rapalink-1, we examined the phosphorylation of S6 at Ser240/244 and Akt at Ser473, respectively. The ANOVA values were as follows: pAkt [*F*_(3, 30)_ = 10.85], pS6 [*F*_(3, 29)_ = 18.78], and MMP2 [*F*_(3, 30)_ = 41.03]. At 2 h after 1 h of MCAO, in the control rats, there was a significant increase in the phosphorylated S6 and Akt in the IR-C compared with the CC (*p* < 0.0001). Rapalink-1 treatment significantly decreased phosphorylated S6 and Akt in the IR-C compared with the control rats (*p* < 0.0001). The MMP2 protein level was significantly increased with Rapalink-1 treatment in the IR-C, and it was higher than the control group (*p* < 0.0001) (Figure 4).

## Discussion

This study demonstrated that Rapalink-1 increased infarct size and BBB disruption in early cerebral ischemia–reperfusion. The phosphorylation of Akt (Ser473) and the phosphorylation of S6 (Ser240/244) became lower in the IR-C with Rapalink-1 compared with the control rats, which suggests that the mTORC2 and mTORC1 activity was inhibited by Rapalink-1, respectively. A higher K_i_ of ^14^C-AIB and a higher level of MMP2 with Rapalink-1 in the IR-C indicate that BBB disruption was aggravated by Rapalink-1 in cerebral ischemia–reperfusion. This study is the first one to show that Rapalink-1, which inhibited both mTORC1 and mTORC2 in the IR-C, could be detrimental to neuronal survival in the first few hours of cerebral ischemia–reperfusion within the time window of thrombolysis therapy.

Rapalink-1 inhibited mTORC1 in the IR-C that was evidenced by decreased pS6 (Ser240/244) compared with the control rats. The inhibition of S6K1 is known to activate PI3K/Akt through a negative feedback loop (Kim et al., 2017), and the activation of PI3K/Akt may be needed for neuronal survival in cerebral ischemia–reperfusion as shown in our previous study (Weiss et al., 2018). mTORC1 is also involved in cell survival through the activation of S6K1-pS6 and antiapoptotic protein Bcl-2 (Shi et al., 2011; Kim et al., 2017; Murugan, 2019). However, Rapalink-1 is a bivalent compound that combines the durable effects of rapamycin and dual inhibition of mTORC1/mTORC2. It blocks the phosphorylation of mTORC1 targets including p-4EBPI and inhibits mTORC2 signaling (Rodrik-Outmezguine et al., 2016; Fan et al., 2018; La Manna et al., 2020). It also crosses the BBB (Fan et al., 2018; Morisot et al., 2018). In our previous study, a high dose of rapamycin increased the cerebral infarct in early cerebral ischemia where both mTORC1 and mTORC2 were blocked. Our current data with Rapalink-1 confirm that blocking both mTORC1 and mTORC2 is detrimental to neuronal survival in the first few hours of cerebral ischemia–reperfusion.

Rapalink-1 inhibited mTORC2 in the IR-C that was evidenced by decreased pAkt (Ser473) compared with the control rats. mTORC2 signaling is sensitive to nutrient and energy fluctuations. The addition of growth factors to serum-starved cells robustly increases the phosphorylation of Akt (Ser473), a hallmark of the activation of mTORC2. The activation of mTORC2 is also increased by acute energy stress and glutamine starvation (Moloughney et al., 2016; Kazyken et al., 2019). In our previous studies, we reported an increase in the mTORC1 and mTORC2 activity in cerebral ischemia–reperfusion where there is a shortage of energy and an imbalance between energy demand and metabolism (Chi et al., 2016a, 2020; Weiss et al., 2018). In this study, the phosphorylation of Akt (Ser473) is increased during ischemia–reperfusion of the brain of the rat, which is consistent with the role of mTORC2 in responding to starvation conditions and neuronal survival through FOX03a (Brunet et al., 2001; Szwed et al., 2021). The elevated phosphorylation of Akt (Ser473) during ischemia–reperfusion is accompanied by increased mTORC1 signaling. Our current data suggest that the activation of mTORC1 and mTORC2 is necessary for neuronal survival.

The enlarged infarct size was noted with Rapalink-1 in the first few hours of ischemia–reperfusion. Our current data suggest that inhibiting both mTORC1 and mTORC2 by Rapalink-1 in early cerebral ischemia could be detrimental to neuronal survival. No other study is currently available about the relationship between Rapalink-1 and neuronal survival in cerebral ischemia. The addition of Rapalink-1 resulted in more than 50% decrease in the phosphorylation of both Akt and S6 in the IR-C when compared with the control rats, which is consistent with the portrayal of the literature of Rapalink-1 as a dual mTOR inhibitor. It is not clear why this inhibition was not present in the CC of the Rapalink-1-treated rats. It is possible that the dose of Rapalink-1 was insufficient to affect mTOR activity in the non-ischemic cortex or the intervals after drug administration might affect the results (Weiss et al., 2018).

Our data indicate an increase in BBB disruption with Rapalink-1 when both mTORC1 and mTORC2 are inhibited in the IR-C. We did not expect an increase in BBB disruption with Rapalink-1 since mTOR is involved in pathways that increase BBB permeability through VEGF, HIF-α, and MMPs (Land and Tee, 2007; Shen et al., 2018; Murugan, 2019). However, Rapalink-1 might affect other mechanisms controlling BBB permeability.

There were some regional differences in K_i_ and the volume of dextran distribution in the non-ischemic brain regions with Rapalink-1 treatment. There is less leakage through the BBB in normal conditions except in the circumventricular areas (Chi et al., 1992; Wilhelm et al., 2016). However, systemic studies are needed to understand the mechanisms of the regional differences in the BBB permeability with various agents such as Rapalink-1. The volume of dextran distribution represents dextran in the plasma as well as dextran that is leaked into brain tissue. Our data suggest that the plasma volume in the CH could be lower than the pons with Rapalink-1 treatment.

^14^C-α-aminoisobutyric acid is a small inert hydrophilic molecule (104 Da). In our data, only K_i_ was increased in the IR-C by Rapalink-1, suggesting that the transfer of ^14^C-AIB across BBB was increased. But the volume of dextran distribution measured by a larger molecule, i.e., dextran, was not significantly altered. Even at this degree of BBB disruption, ions, neurotransmitters, chemicals, and toxins could cross the BBB and could aggravate neuronal damage in cerebral ischemia–reperfusion. Rapalink-1 increased K_i_ in both the ischemic-reperfused and the contralateral cortices. Our current data suggest that Rapalink-1 may have affected the baseline BBB permeability in all other brain regions, not just in the ischemic-reperfused area. Rapalink-1 may have produced the systemic effects in addition to intracellular signaling to affect BBB permeability, or Rapalink-1 at this dose could not suppress postischemic inflammation, which increased BBB permeability (Xin et al., 2015; Yang et al., 2021).

The mechanism is not clear, but in the IR-C, the MMP2 protein level was significantly increased by Rapalink-1 compared with the control rats. The higher MMP2 suggests an increase in degradation of extracellular matrix such as tight junction or basal lamina, leading to an increase in the BBB disruption. This higher MMP2 could have contributed to the aggravation of BBB disruption by Rapalink-1 in the IR-C in this study.

## Conclusions

This study demonstrated that Rapalink-1 produced the inhibition of both mTORC1 and mTORC2 in the early stage of cerebral ischemia–reperfusion that is within the time window of thrombolysis therapy. It increased infarct size and BBB disruption. Taken together, our data suggest that inhibiting both mTORC1 and mTORC2 by Rapalink-1 could worsen the neuronal damage in the early stage of cerebral ischemia–reperfusion and that the aggravation of BBB disruption could be one of the contributing factors.

## Data Availability Statement

The raw data supporting the conclusions of this article will be made available by the authors, without undue reservation.

## Ethics Statement

The animal study was reviewed and approved by Institutional Animal Care and Use Committee, Rutgers Robert Wood Johnson Medical School.

## Author Contributions

OC: conceptualization, formal analysis, visualization, writing original draft, and data curation. XL: conceptualization, methodology, investigation, and data curation. SC: investigation and reviewing and editing the draft. NP: visualization, methodology, data curation, and formal analysis. EJ: funding acquisition, conceptualization, formal analysis, methodology, and reviewing and editing the draft. HW: conceptualization, software, validation, formal analysis, and reviewing and editing the draft. All authors contributed to the article and approved the submitted version.

## Conflict of Interest

The authors declare that the research was conducted in the absence of any commercial or financial relationships that could be construed as a potential conflict of interest.
